# Effect of Ce Doping on the Structure and Chemical Stability of Nano-α-Fe_2_O_3_

**DOI:** 10.3390/nano9071039

**Published:** 2019-07-21

**Authors:** Junxiang Ning, Peiyang Shi, Maofa Jiang, Chengjun Liu, Xiaoliang Li

**Affiliations:** 1 Key Laboratory for Ecological Metallurgy of Multimetallic Ores (Ministry of Education), School of Metallurgy, Northeastern University, Wenhua Road, Heping District, Shenyang 110819, China; 2 School of Metallurgical Engineering, Liaoning Institute of Science and Technology, Liaoning, Fragrance of huaihe road, High-tech Industrial Development District, Benxi 117004, China

**Keywords:** Ce-doping, nano-α-Fe_2_O_3_, composite, chemical stability

## Abstract

Ce-doped nano-α-Fe_2_O_3_ was successfully synthesized via the hydrothermal method. The properties of the prepared particles were studied by X-ray diffraction (XRD), scanning electron microscope (SEM), transmission electron microscopy (TEM), Fourier transform infrared (FTIR), X-ray photoelectron spectroscopy (XPS) and electrochemical methods. It was found that the Ce element can be doped into the α-Fe_2_O_3_ lattice resulting in lattice distortion, which can refine the grain and improve the crystal surface’s integrity significantly. In addition, doping of Ce element can shorten the Fe–O bond length in the α-Fe_2_O_3_ crystal, cause a blue shift of the stretching vibration band, enhance binding energy of Fe–O and the chemical stability of the α-Fe_2_O_3_ crystal.

## 1. Introduction

With the development of science and technology, nano-materials have played an important role in various fields with their unique properties [1,2]. Nano-iron oxide is widely used in ships, power facilities and automobiles due to its excellent hiding power, color strength and chemical stability [3,4,5,6,7]. It is well known that when a metallic coating is in contact with electrolytes for a long time, corrosive ions will penetrate the surface of the substrate through the coating. Once oxygen-consuming corrosion occurs at the coating/electrolyte interface, electrochemical corrosion of small anodic coupled with large cathodic will begin. Corrosion reaction will inevitably deteriorate the stability of the coating and reduce its corrosion resistance [8,9]. Therefore, the development of high-performance coatings has received considerable attention [10,11]. If the physical barrier and chemical stability of nano-iron oxide are improved and applied to the field of high-performance coating materials, it will inevitably exert a profound influence on the properties of the coating materials. However, the related reports are very limited.

Liu and Sun [12] found that CeO_2_ doped could improve the chemical activity of Fe_2_O_3_/γ-Al_2_O_3_ nanoparticles. Without changing the valence state of Fe^3+^, the surface of the nanoparticles adsorbs oxygen, thereby improving its chemical properties. It was worth noting that Wang et al [13]. Substituted Fe in α-Fe_2_O_3_ structure by Ce to form a solid solution. The ionic radius of Fe^3+^ is smaller than that of Ce^4+^, resulting in an increase in the lattice constant of Fe_2_O_3_, oxygen vacancy concentration and specific surface area. Tambe et al. [14] and Singh et al. [15] found that the concentration of iron oxide can affect the mechanical properties and corrosion resistance of coatings. Sathiyanarayanan et al. [16] used chemical oxidation to prepare polyaniline-Fe_2_O_3_ composite materials and found that it had a better corrosion resistance than an ordinary Fe_2_O_3_ coating. Shailesh and Khanna [17] studied the effect of nano-iron oxide on the optical, mechanical and corrosion behavior of coatings. The results showed that adding one small amount of nano-iron oxide could improve the corrosion resistance, UV resistance and abrasion resistance of the coatings. Although the ordinary nano-iron oxide can block the contact of water, oxygen, and corrosive ions in the electrolyte solution with the substrate, the protective ability of nano-iron oxide on the metal surface is limited due to its structure and properties. For example, conventional nano-iron oxide coatings do not have passivation corrosion inhibition. Once the coating is corroded, it will not repair the crack. In order to improve the performance of nano-iron oxide, related research mostly focus on the morphology and particle size control of nano-iron oxide. Huo et al. [18] utilize hydrothermal methods to prepare nano-rods α-FeOOH and fusiform α-Fe_2_O_3_ according to different NaOH concentrations. Liu et al. [19] used the hydrothermal method to prepare nano-N-rGO/Fe_2_O_3_ composites and found that the Fe_2_O_3_ particles doped with nitrogen had a smaller size and stronger electrochemical performance than that of ordinary Fe_2_O_3_.

Although the research results of the above scholars had a certain effect on improving the electrochemical stability of iron oxide, it is not sufficient to change the shape or reduce the particle size of the nano-iron oxide particles to enhance their performance, which still cannot satisfy the application of the current harsh environment. In this paper, pure and Ce-doped Fe_2_O_3_ were prepared by hydrothermal method using ceric sulfate and ferric sulfate as raw materials. We analyzed the influence of Ce element on the microstructure and electrochemical stability of Fe_2_O_3_. The pure and Ce-doped Fe_2_O_3_ was studied by using X-ray diffraction (XRD), scanning electron microscope (SEM), transmission electron microscopy (TEM), Fourier transform infrared (FTIR), X-ray photoelectron spectroscopy (XPS) and electrochemical methods. Finally, we used Materials Studio (MS) software to simulate the structure of Fe_2_O_3_ crystal lattice after doping Ce element, and visually show the influence and existence of Ce element in the Fe_2_O_3_ crystal lattice.

## 2. Experimental Procedure

### 2.1. Raw Materials

Ce(SO_4_)_2_, Fe_2_(SO_4_)_3_, NaCl, NaOH, H_2_SO_4_ (AR; Sino-Pharm Chemical Reagent Co., Ltd., Shanghai, China). Nano-iron oxides produced by the Bayer Company (Shanghai, China) were chosen as the comparative sample.

### 2.2. Preparation of Nano-Iron Oxide

The Ce-doped α-Fe_2_O_3_ was prepared by hydrothermal reactions of 0.0099 M cerium (IV) sulfate solutions and 0.5 M ferric sulfate solutions in 250 mL Teflon-lined stainless steel autoclave. The pH of the solutions was adjusted to about 5, with the addition of 2 M NaOH. The autoclave was sealed and maintained at 160 °C for 1 h. After that, the final solid products were collected by filtration, washed with large amounts of deionized water and ethanol to remove all impurities, and dried at 40 °C for 10 h under vacuum. Finally, the Ce-doped α-Fe_2_O_3_ was obtained by subsequent calcination at 700 °C in a muffle furnace for 1 h. The undoped α-Fe_2_O_3_ preparation process was the same as the Ce-doped α-Fe_2_O_3_ preparation process, except that no cerium (IV) sulfate was added.

### 2.3. Characterization

(1) XRD analysis. The crystal structure was measured by using X-ray diffraction (XRD) (PANalytical, X’pert PRO, Almelo, Netherlands) with Cu K_α_ (*λ* = 1.5418 Å). The scan rate and 2*θ* range of the samples were 2 deg/min and 10–70°, respectively. Moreover, the crystal structure was analyzed with jade6 software (Materials Data Inc., California, CA, USA) and in accordance with the databases of Powder Diffraction Flile (PDF) provided by the International Centre for Diffraction Data (ICDD). The crystalline size of nano-iron oxide can be estimated from the full width at half-maximum diffraction peak by the Scherrer equation.
  (1) D=Kλ/(βcosθ)
  where *D* is the crystalline size (nm), *K* is a geometric factor (use *K* = 0.9), *λ* is the X-ray wavelength (1.5418 Å), *β* is the peak full width at half maximum (FWHM) in radians and *θ* is the Bragg’s angle of the peaks.
(2) SEM-EDS analysis. The samples were first sprayed gold and then investigated for their morphology by scanning electron microscopy (SEM) (Zeiss, ULTRA PLU, Oberkochen, Germany).
(3) TEM analysis. Transmission electron microscopy (TEM) (JEOL, JEM 2100, Tokyo, Japan) was used to study the Ce-doped α-Fe_2_O_3_’s internal phase at 200 keV.
(4) FTIR analysis. The bonding styles of samples were studied using Fourier transform infrared (FTIR) (Thermo Nicolet, Nicolet-380, Minneapolis, MN, USA) in the 4000–400 cm^−1^ range using the KBr dilution technique.
(5) XPS analysis. The element type and chemical valence state of the sample surface was measured by using the American Thermo Scientific ESCALAB 250Xi electron spectrometer (Waltham, MA, USA).
(6) Electrochemistry analysis. Potentiodynamic polarization measurements were performed with an electrochemical workstation (Metrohm, Autolab, Utrecht, Switzerland). The scan rate was 3 mV s^−1^_,_ with the scanning potential ranging from −0.5 to +1 V (vs. OCP). Samples, liquid paraffin and carbon powder were mixed in a 1:4:5 mass ratio as working electrode [20], a platinum plate (Pt) was used as the counter electrode and a saturated calomel electrode (SCE) was the reference electrode. The measurement was performed at room temperature in a 3.5% NaCl solution. The analysis was carried out using the NOVA 1.10 software (Metrohm, Beijing, China) provided by the Autolab electrochemical workstation.

## 3. Results and Discussion

**Figure 1:**
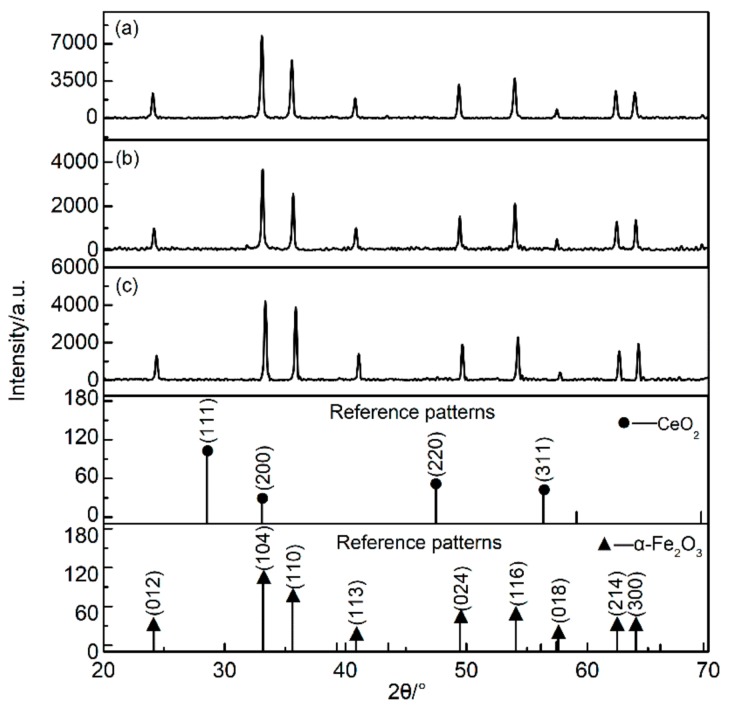
XRD (X-ray diffraction) pattern of the samples. (**a**) Ce-doped α-Fe_2_O_3_ sample; (**b**) undoped α-Fe_2_O_3_ sample; (**c**) Bayer α-Fe_2_O_3_ sample.

**Figure 2:**
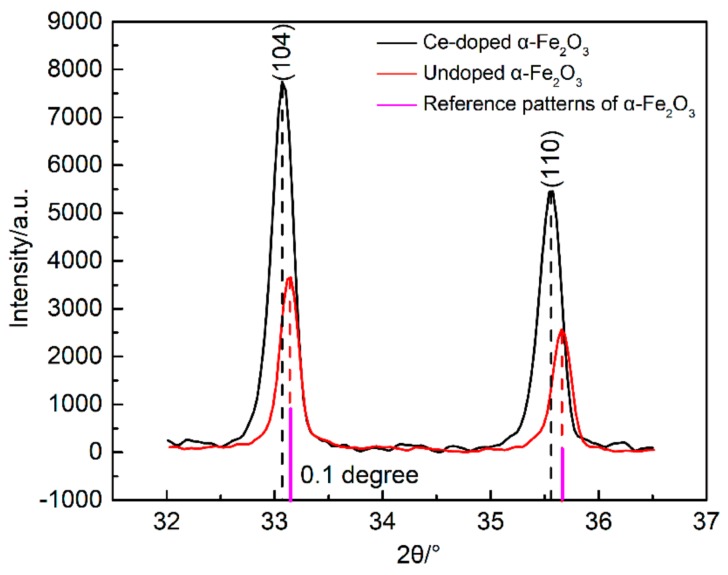
Comparison of (104) and (110) peaks from the XRD patterns.

Figure 1 shows the XRD curve of the sample. It can be seen that the three samples were all hexagonal crystal structure of α-Fe_2_O_3_ (JCPDS 33-0664) with good crystallinity. No characteristic peaks of the CeO_2_ were discovered in the Ce-doped α-Fe_2_O_3_ sample, which suggests that the Ce element enters into the α-Fe_2_O_3_ lattice [21,22]. In the Ce-doped α-Fe_2_O_3_ crystal phase, the intensity of the three characteristic peaks (104), (110) and (116) was obviously enhanced and the three peaks get broader. The average crystal size of the samples was determined from the broadening of the diffraction peaks (104), (110) and (116) plane using Scherrer’s equation. The results for average particle size, the lattice constant, and half-width of the characteristic peak of the three Nano samples can be obtained from Table 1. The (104), (110) and (116) characteristic peaks of the Ce-doped α-Fe_2_O_3_ were wider than that of the undoped α-Fe_2_O_3_ and Bayer α-Fe_2_O_3_, which indicates the Ce-doped α-Fe_2_O_3_ had a smaller particle size. Since the Ce^4+^ radius (1.01Å) [23] is larger than the Fe^3+^ radius (0.64 Å) [23], when Ce^4+^ enters the lattice, the lattice constant increases and the characteristic peak broadens. Therefore, it is considered that the doping of the Ce element causes lattice distortion of α-Fe_2_O_3_ [24]. In order to investigate the effect of doping on the phase structures of the α-Fe_2_O_3_ particles, the (104) and (110) diffraction peaks were monitored. Figure 2 displays that there was a 0.1° left shift in (104) and (110) diffraction peaks of Ce-doped α-Fe_2_O_3_. All of the results proved that the incorporation of Ce ions led to lattice deformation without deteriorating the original crystal structure.

**Figure 3:**
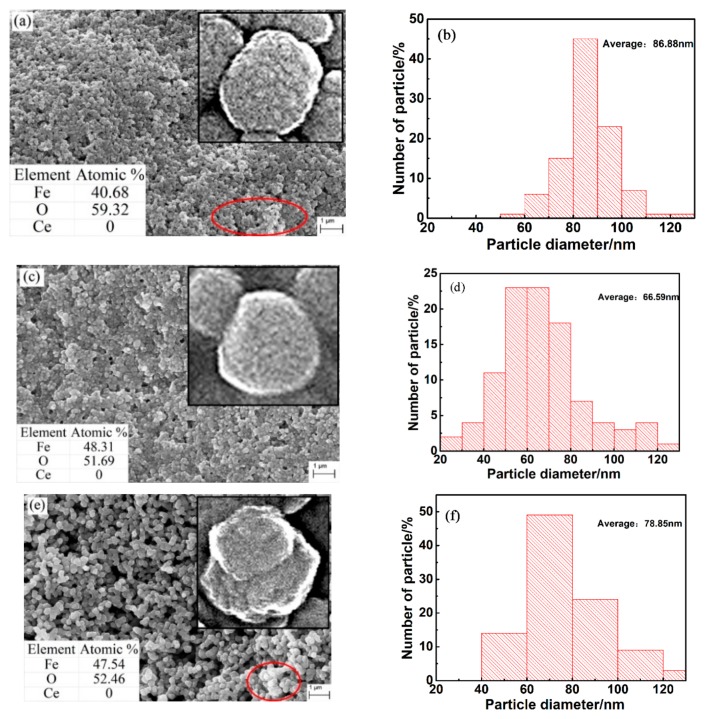
SEM (scanning electron microscopy) images and size distribution histograms of samples. (**a**,**b**) Undoped α-Fe_2_O_3_ sample; (**c,d**) Ce-doped α-Fe_2_O_3_ sample; (**e,f**) Bayer α-Fe_2_O_3_ sample.

Figure 3 shows the microscopic morphology of the three nano-iron oxides. Obvious agglomeration can be observed in the undoped α-Fe_2_O_3_ and Bayer α-Fe_2_O_3_, the particles in the Ce-doped α-Fe_2_O_3_ sample have high dispersibility. Nano Measure1.2.0 software was used to calculate the particle size of random 100 particles in three fields of three samples. The histograms in Figure 3 show the average size distribution of particles of the three samples. The average particle sizes were 87, 67 and 79 nm for the samples of undoped α-Fe_2_O_3_, Ce-doped α-Fe_2_O_3_ and Bayer α-Fe_2_O_3_, respectively. In the Ce-doped α-Fe_2_O_3_ sample, the distribution of particle size is consistent and the dimension distribution of powder is narrow. These results are similar to the results of XRD in Table 1. At the same time, the surfaces of the undoped α-Fe_2_O_3_ and Bayer α-Fe_2_O_3_ were rough while the surfaces of Ce-doped α-Fe_2_O_3_ appeared smoother. According to the analysis of EDS, the iron-oxygen ratio was 1:1.46, 1:1.07 and 1:1.10 for the samples of undoped α-Fe_2_O_3_, Ce-doped α-Fe_2_O_3_ and Bayer α-Fe_2_O_3_, respectively. The iron-oxygen of undoped α-Fe_2_O_3_ in this paper conformed to the standard iron oxide. Ce-doped α-Fe_2_O_3_ was detected by EDS, and it was found that the atomic ratio of Fe and O in Fe_2_O_3_ was not the standard 1:1.5, which may be caused by the doping of Ce element.

**Figure 4:**
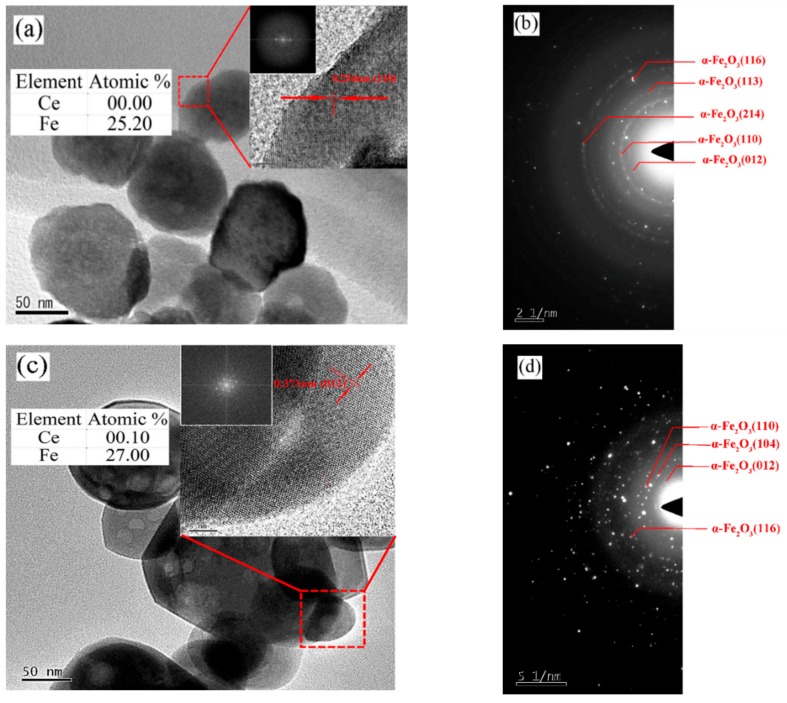

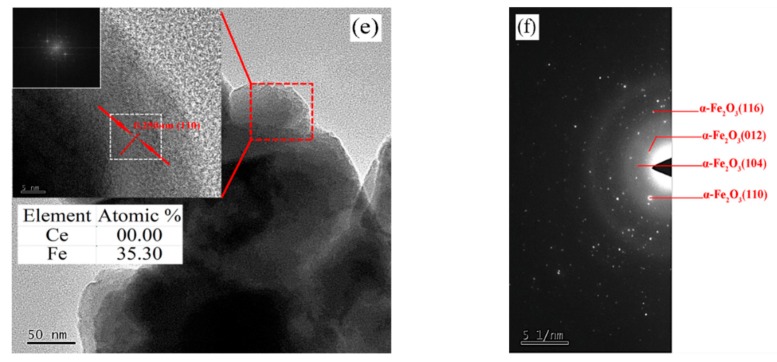
TEM (transmission electron microscopy) images of samples. (**a,b**) Undoped α-Fe_2_O_3_ sample; (**c,d**) Ce-doped α-Fe_2_O_3_ sample; (**e,f**) Bayer α-Fe_2_O_3_ sample.

Transmission electron microscopy (TEM) and high-resolution TEM (HRTEM) images provide further insight into the microstructure and morphology of the three nano-iron oxides. Figure 4a shows a high-magnification TEM image of undoped α-Fe_2_O_3_ with a coarse surface. Similar to the results of SEM, there was agglomeration between the particles. The HRTEM images inset in Figure 4a further confirm the ellipsoid structure of undoped α-Fe_2_O_3_ particles. The fringes in a typical HRTEM image were separated by ~0.254 nm, which agrees well with the {110} plane of the α-Fe_2_O_3_. The selected area electron diffraction (SAED) pattern shown in Figure 4b was obtained from the polycrystalline structure of undoped α-Fe_2_O_3_, which is in agreement with the XRD results. Figure 4c shows the morphology of the regular polygonal structure of Ce-doped α-Fe_2_O_3_ with a complete boundary, a smooth surface and well-dispersed. The HRTEM images inset of Figure 4c show a d value of ~0.371 nm, which corresponds to {012} of α-Fe_2_O_3_. The SAED pattern of Ce-doped α-Fe_2_O_3_ shows one kind of spotty ring patterns of α-Fe_2_O_3_ (JCPDS 33-0664). The above experimental results demonstrate again that the Ce element was doped into the α-Fe_2_O_3_ lattice, causing the expansion of the α-Fe_2_O_3_ lattice and the increase of the interplanar spacing. The degree of surface roughness and agglomeration of Bayer α-Fe_2_O_3_ particles (Figure 4e) was the most severe among the three samples.

In this paper, it is considered that Ce doping into α-Fe_2_O_3_ crystal resulted in inhibiting the growth of iron oxide crystals. According to the RIGGI mechanism proposed by Kools [25], doping Ce element into the interior of α-Fe_2_O_3_ lattice results in a decrease in free energy, thereby the growth of grain is suppressed. This demonstrates that Ce doping not only affects the surface activity of α-Fe_2_O_3_ grains but also affects the state of grain growth, this phenomenon has also been mentioned in other studies [12].

**Figure 5:**
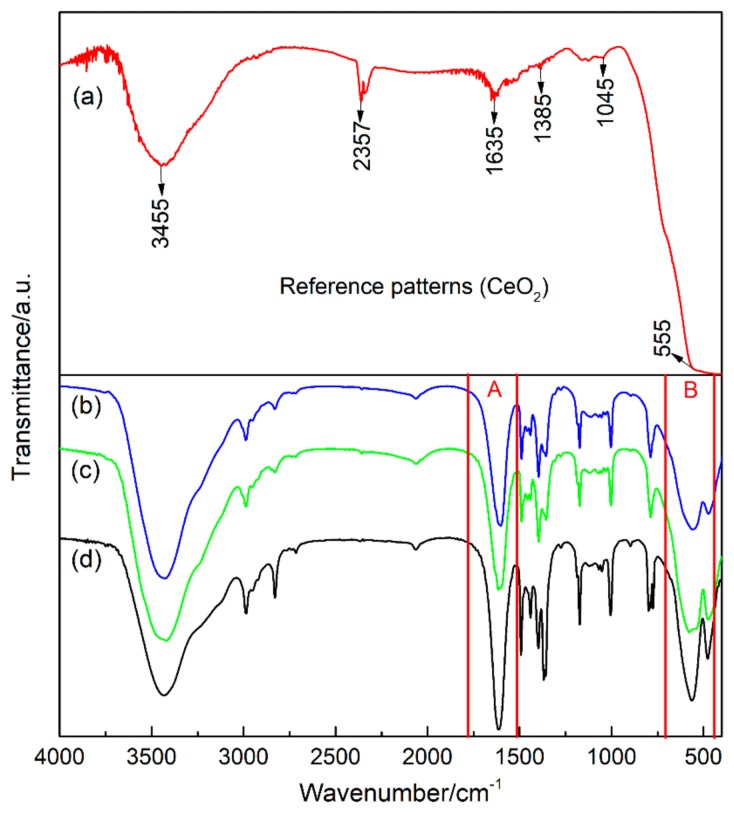
FTIR (Fourier transform infrared) spectra of samples. (**a**) pure CeO_2_; (**b**) undoped α-Fe_2_O_3_ sample; (**c**) Ce-doped α-Fe_2_O_3_ sample; (**d**) Bayer α-Fe_2_O_3_ sample.

Figure 5 shows the FTIR spectra of the three nano-iron oxides. It can be seen from Figure 5a that the infrared absorption bands appeared in pure CeO_2_ crystals at 3455 cm^−1^, 2357 cm^−1^, 1635 cm^−1^, 1385 cm^−1^, 1045 cm^−1^ and 555 cm^−1^. The absorption bands at 3455 cm^−1^, 1385 cm^−1^ and 1045 cm^−1^ were stretching vibration bands and the bending vibration bands of the water are absorbed by the CeO_2_ powder [26]. The infrared absorption band around 2357 cm^−1^ was due to CO_2_ which was absorbed from the atmosphere [27]. The infrared absorption bands around 1635 cm^−1^ and 555 cm^−1^ were attributed to the asymmetric stretching vibration band and symmetrical vibration band of Ce–O [26,28], respectively. Generally, metal oxides exhibit an infrared absorption band between 400 cm^-1^ and 675 cm^−1^ [29].

**Figure 6:**
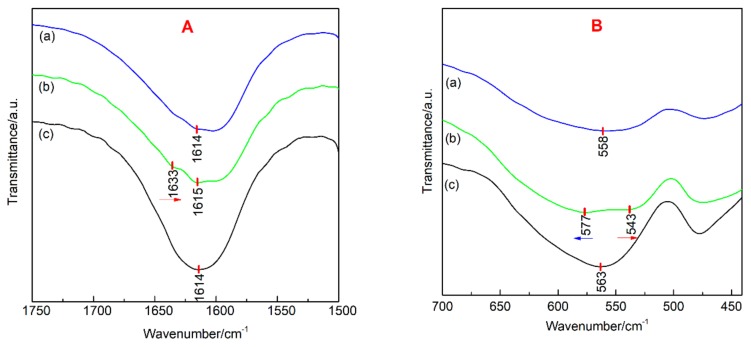
FTIR spectra local amplification results of samples (A and B are enlarged view of Figure 5.). (**a**) Undoped α-Fe_2_O_3_ sample; (**b**) Ce-doped α-Fe_2_O_3_ sample; (**c**) Bayer α-Fe_2_O_3_ sample.

Figure 6 shows an enlarged view of region A and B of the FTIR spectra in Figure 5. The A region is an infrared absorption band between 1750 cm^−1^ and 1500 cm^−1^, and the B region is an infrared absorption band between 700 cm^−1^ and 450 cm^−1^. In the A region, it was found that the three kinds of samples showed H–O–H stretching vibration bands near 1600 cm^−1^, and the asymmetric stretching vibration band of Ce–O of Ce-doped α-Fe_2_O_3_ appear at 1633 cm^−1^, this is consistent with the results of Ishaque et al [30]. Compared with the FTIR results of pure CeO_2_ crystals, a red shift of 2 cm^−1^ of Ce–O occurred in Ce-doped α-Fe_2_O_3_. The addition of surfactant, resulted in an increase in surface water absorption of Bayer α-Fe_2_O_3_ so that the water molecules’ stretching vibration bands showed a significantly blue shift. In the B region, it was found that a symmetrical vibration band of Ce–O appeared at 543 cm^−1^ in the Ce-doped α-Fe_2_O_3_, which had a red shift of 12 cm^−1^ compared with the pure CeO_2_. The infrared absorption band of Fe–O of undoped α-Fe_2_O_3_ appeared at 558 cm^−1^, and the infrared absorption band of Fe–O of Ce-doped α-Fe_2_O_3_ appeared at 577 cm^−1^. Therefore, the Fe–O stretching vibration band in Ce-doped α-Fe_2_O_3_ crystals underwent blue shifts of 9 cm^−1^. The infrared absorption bands were considerably wider in Ce-doped α-Fe_2_O_3_ than in undoped α-Fe_2_O_3_.

The essence of the infrared absorption process is the resonance absorption of incident infrared light by a molecular vibration system resulting in a transition of energy levels. The molecular vibration frequency is shown in Equation (2):

(2) $$v=\frac{1}{2\pi \cdot c}\sqrt{\frac{k}{\mu }}$$

where *c* is the speed of light, *k* is the constant of the force constant of a chemical bond, μ is equivalent to the relative atomic mass.

In the Ce-doped α-Fe_2_O_3_ crystals, the main reason for the blue shift of the Fe–O stretching vibration bands in Ce-doped α-Fe_2_O_3_ is the quantum size effect [31]. Ce doping causes the lattice of α-Fe_2_O_3_ expansion and increases the lattice constant. Lattice distortion causes a decrease in average bond length of the Fe–O bonds, while the chemical bond constant *k*, bond energy and the frequency of the vibration bond increase, and the Fe–O bond blue shift. The quantum size effect also causes the fine band structure of the original conventional bulk material of the nanoparticles to disappear, resulting in a broadening of the infrared absorption band [32,33].

**Figure 7:**
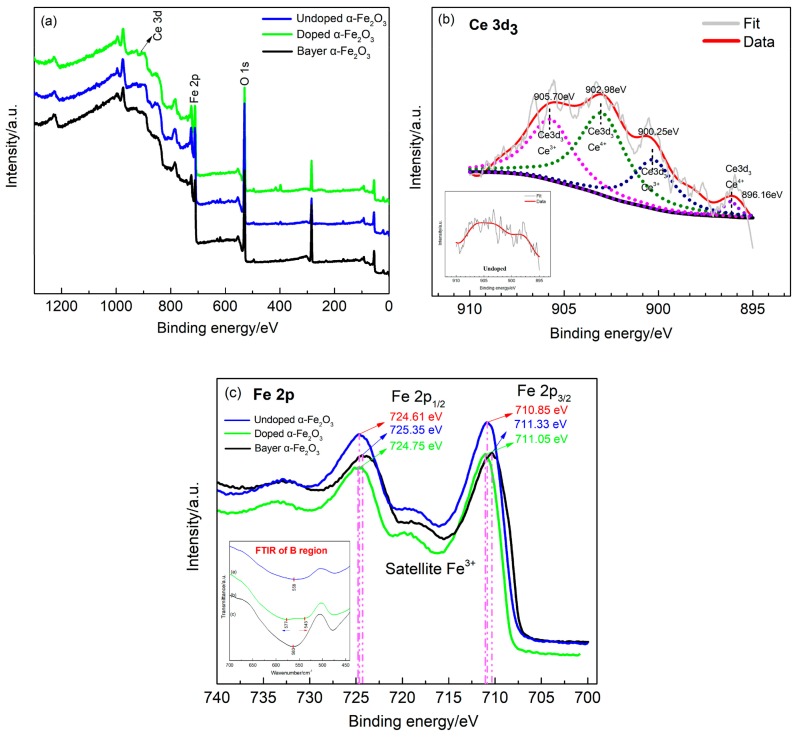
Survey XPS (X-ray photoelectron spectroscopy) spectrum of samples. (**a**) Representative XPS survey spectrum of three samples; high resolution XPS spectra of (**b**) Ce 3d; (**c**) Fe 2p.

The chemical composition of the three nano-iron oxides was also studied by XPS analysis. According to Figure 7a, the overall XPS survey of Ce-doped α-Fe_2_O_3_ includes O 1s, Ce 3d and Fe 2p. However, the XPS survey of the other two samples only includes O 1s and Fe 2p. In order to determine the presence of various components in the three samples, the Ce 3d and Fe 2p peaks were deconvoluted using a peak fitting process (Figure 7b,c). Figure 7b exhibits the Ce 3d spectrum was separated four peaks at binding energies of 896.16 eV, 900.25 eV, 902.98 eV and 905.70 eV, verifying that the cerium was available in two oxidation states of Ce^3+^ and Ce^4+^ [34,35]. It can be seen that the intensity of the peaks related to Ce^4+^ is greater than Ce^3+^, which can prove a fact that cerium was mostly in the form of Ce^4+^ in the α-Fe_2_O_3_ lattice. However, these peaks of Ce 3d of undoped α-Fe_2_O_3_ were not visible inset Figure 7b. The high-resolution Fe 2p peaks of the three samples are depicted in Figure 7c. The binding energies of Fe 2p_1/2_ and Fe 2p_3/2_ of three samples were located in 710 eV–726 eV, which is in good agreement with the earlier reports [36]. Moreover, a satellite peak was observed in the Fe 2p spectrum of three samples, which is characteristic of Fe_2_O_3_. More importantly, it was observed that the binding energy of outer electron of Fe^3+^ of Ce-doped α-Fe_2_O_3_ was larger than that undoped α-Fe_2_O_3_. It indicated that the Ce element was doped into the crystal lattice and enhanced the bond energy of Fe and O, which was completely consistent with the FTIR results inset in Figure 7c.

**Figure 8:**
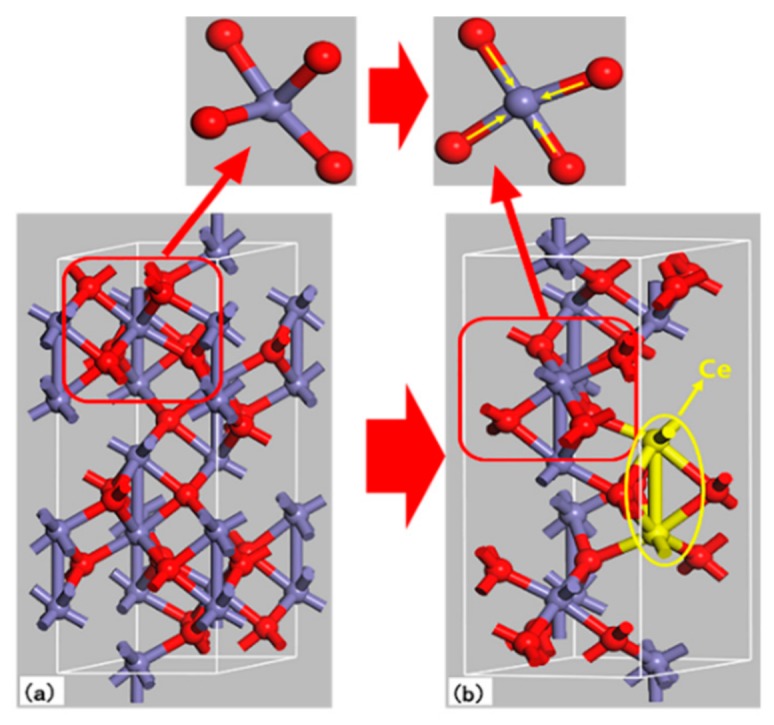
Unit cell of α-Fe_2_O_3_ (purple ball is O; red ball is Fe; yellow ball is Ce). (**a**) Undoped α-Fe_2_O_3_ sample; (**b**) Ce-doped α-Fe_2_O_3_ sample.

Figure 8 is a model of α-Fe_2_O_3_ crystal structure simulated by Materials Studio software. When Ce elements are doped into the α-Fe_2_O_3_ crystal, Ce^4+^ will replace the position of the Fe^3+^ and bond with O^2−^. Ce atoms exist in the form of Ce^4+^ in the α-Fe_2_O_3_ lattice, which could donate one free electron in the α-Fe_2_O_3_ lattice [37]. Therefore, it is considered that the doping of Ce reduces the Fe–O bond length and increases the bond energy, which enhances the crystal stability [38,39].

**Figure 9:**
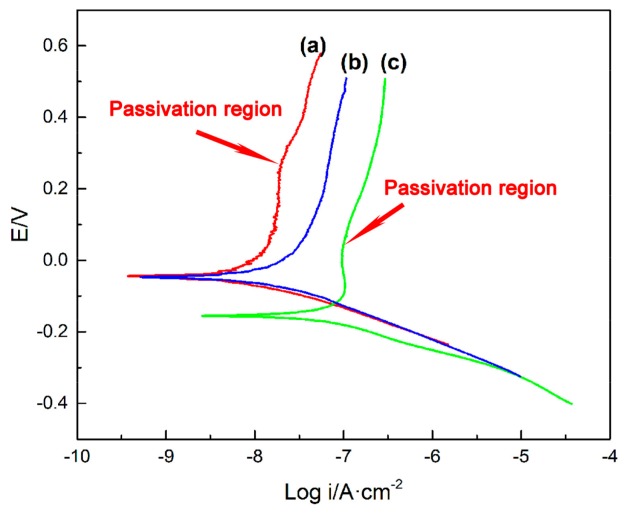
Electrochemical test of samples. (**a**) Ce-doped α-Fe_2_O_3_ sample; (**b**) Bayer α-Fe_2_O_3_ sample; (**c**) undoped α-Fe_2_O_3_ sample.

Figure 9 shows the potentiodynamic polarization curves of the three samples. Corrosion potentials (*E*_corr_), corrosion currents (*I*_corr_) and breakdown potential (*E*_brea_) are given in Table 2. In general, the higher the *E*_corr_, the better the chemical stability, the smaller the *I*_corr_, and the smaller the corrosion rate. The higher the *E*_brea_, the more difficult it is to form pitting. According to Table 2, the *E*_corr_ of Ce-doped α-Fe_2_O_3_ (−44.08 mV) was higher than that of undoped α-Fe_2_O_3_ (−155.12 mV). The *I*_corr_ of e-doped α-Fe_2_O_3_ (0.01 A·cm^−2^) was lower than that of undoped α-Fe_2_O_3_ (0.12 A·cm^−2^) and the *E*_brea_ of Ce-doped α-Fe_2_O_3_ (252.23 mV) was higher than that of undoped α-Fe_2_O_3_ (29.37 mV). The results show that the Ce-doped α-Fe_2_O_3_ particles can improve the electrochemical stability of the particles and promote the passivity of the nanoparticles. Compared with the *E*_corr_ (−46.46 mV) and *I*_corr_ (0.03 A·cm^−2^) of Bayer α-Fe_2_O_3_, the *E*_corr_ of Ce-doped α-Fe_2_O_3_ was more positive and the *I*_corr_ was smaller. As shown in Figure 8, the anodic polarization curve of the Bayer α-Fe_2_O_3_ had no obvious passivation region. Therefore, the Ce-doped α-Fe_2_O_3_ prepared under this experimental condition was more stable than the Bayer α-Fe_2_O_3_. 

## 4. Conclusions

Under these experimental conditions, nanoparticles of Ce-doped α-Fe_2_O_3_ with regular polygonal structure were prepared by the hydrothermal method, which had good dispersion and chemical stability. The doping of Ce into the α-Fe_2_O_3_ lattice did not alter the original phase composition of α-Fe_2_O_3_. However, the distortion of the α-Fe_2_O_3_ lattice resulted in the decrease of grain size and increase of the degree of dispersion. Doping of Ce can shorten the Fe–O bond length in α-Fe_2_O_3_ crystals and enhance the Fe–O bond energy. The increasing bond energy promoted the blue shift of the Fe–O stretching vibration bands and strengthened the passivity of the surface of the α-Fe_2_O_3_ particle. The electrochemical stability of the α-Fe_2_O_3_ particle was thus improved.

## Figures and Tables

**Table 1:** Average particle size and lattice constant of nanoparticles ^a^.

Sample	Average Particle Size/nm	Lattice Constant/a	Lattice Constant/b	Lattice Constant/c	FWHM (104)/deg
Ce-doped α-Fe_2_O_3_	63.9	0.5042	0.5042	1.3771	0.235
Undoped α-Fe_2_O_3_	82.9	0.5034	0.5034	1.3772	0.188
Bayer α-Fe_2_O_3_	77.0	0.5014	0.5014	1.3713	0.231

**Table 2:** Electrochemical parameters of the Tafel curves of the three nanoparticles in 3.5% NaCl solution ^b^.

Sample	E_corr_/mV	I_corr_/μA·cm^−2^	E_brea_/mV
Ce-doped α-Fe_2_O_3_	−44.08	0.01	252.23
Undoped α-Fe_2_O_3_	−155.12	0.12	29.37
Bayer α-Fe_2_O_3_	−46.46	0.03	-
